# Long-term effects of chloropicrin fumigation on soil microbe recovery and growth promotion of *Panax notoginseng*

**DOI:** 10.3389/fmicb.2023.1225944

**Published:** 2023-07-14

**Authors:** Xin Wang, Qing Wang, Wenjing Li, Daqi Zhang, Wensheng Fang, Yuan Li, Qiuxia Wang, Aocheng Cao, Dongdong Yan

**Affiliations:** State Key Laboratory for Biology of Plant Disease and Insect Pests, Institute of Plant Protection, Chinese Academy of Agricultural Sciences, Beijing, China

**Keywords:** *Panax notoginseng*, fumigation, microbial diversity, normalized stochasticity ratio, microbial community assembly

## Abstract

**Introduction:**

*Panax notoginseng* is a precious Chinese medicinal material. Soil fumigation can control soil-borne disease and overcome the continuous cropping obstacles of *P. notoginseng*. However, chloropicrin (CP) fumigation can kill non-target soil microorganisms and reduce microbial diversity, but the long-time impacts of CP fumigation on soil microbial are less reported.

**Methods:**

We studied the long-term effects of CP fumigation on soil microbes with high-throughput gene sequencing, and correlated the changes in the composition of microbial communities with environmental factors like soil physicochemical properties and soil enzyme activities. This study mainly focuses on the recovery characteristics of soil microbe after soil fumigation by evaluating the ecological restoration of *P. notoginseng* soil, its sustained control effect on plant diseases, and its promotion effect on crop growth by focusing on the CP fumigation treatment.

**Results:**

The results showed that CP fumigation significantly increased soil available phosphorus (P) to 34.6 ~ 101.6 mg/kg and electrical conductivity (EC) by 18.7% ~ 34.1%, respectively. High-throughput gene sequencing showed that soil fumigation with CP altered the relative abundance of *Trichoderma*, *Chaetomium*, *Proteobacteria*, and *Chloroflexi* in the soil while inhibiting a lot of Fusarium and Phytophthora. The inhibition rate of Phytophthora spp. was still 75.0% in the third year after fumigation. Fumigation with CP enhanced *P. notoginseng’s* survival rate and stimulated plant growth, ensuring *P. notoginseng’s* healthy in the growth period. The impact of fumigation on microbial community assembly and changes in microbial ecological niches were characterized using normalized stochasticity ratio (NST) and Levins’ niche breadth index. Stochasticity dominated bacterial community assembly, while the fungal community was initially dominated by stochasticity and later by determinism. Fumigation with CP reduced the ecological niches of both fungi and bacteria.

**Conclusion:**

In summary, the decrease in microbial diversity and niche caused by CP fumigation could be recovered over time, and the control of soil pathogens by CP fumigation remained sustainable. Moreover, CP fumigation could overcome continuous cropping obstacles of *P. notoginseng* and promote the healthy growth of *P. notoginseng*.

## 1. Introduction

*Panax notoginseng*, also known as Sanqi or Tianqi in China, is a precious Chinese medicinal material belonging to the genus Panax of the Araliaceae family (Zhao et al., 2020). It has good effects in preventing cardiovascular diseases and improving blood circulation. *P. notoginseng* is used as raw material for many Chinese traditional medicines (Li et al., 2009) and has a planting history of more than 400 years in Southwest China. Wenshan in China is the world’s central production area of *P. notoginseng* (Fan et al., 2016).

The growth environment of *P. notoginseng,* particularly, and many continuous severe cropping obstacles (Tan et al., 2017), and the incidence of *P. notoginseng* after repeated planting in succession can be as high as 70% or more. The soil microflora changes are the major causes of the obstacles of the continuous cropping of *P. notoginseng* (Guo et al., 2009; Ma et al., 2013). Soil fumigation can break the obstacle of constant cropping and improve the yield and quality of crops. It has been widely used in high-value crops such as *P. notoginseng*, yam, lily, etc. (Yan et al., 2022).

Soil fumigation technology can effectively control soil-borne diseases, nematodes, weeds, and underground pests but also have specific effects on the structure and diversity of non-target microbial communities in the soil (Dangi et al., 2015). The impact of soil fumigation on microbial diversity and community structure varied with fumigant kinds and soil types (Fang et al., 2020). CP fumigation reduced the diversity of the soil bacterial population (Li J. et al., 2022). Within 2 months, the variety and abundance of bacteria and fungi reached their pre-fumigation levels (Fang et al., 2020). After Fumigation, the recovery of beneficial microbial like *Mortierella* and *Sphingomonas* is aided by applying organic fertilizer, microbial fertilizer, and humic acid (Cheng et al., 2021; Li Q. et al., 2022). However, the long-time impacts of CP fumigation on soil microbial are less reported.

Soil microbes play a significant role in the geochemical cycle of nutrients, organic matter (OM) breakdown (Tebo et al., 2005), pesticide degradation (Rodriguez et al., 2020), and other activities in the soil ecosystem. They can also be utilized as markers for assessing the health and fertility of the soil (Rashid et al., 2016). Microbial populations become chaotic under environmental stress (Santos-Medellin et al., 2021). The neutral community model (NCM), RCBray, beta nearest taxon index (βNTI), and NST are commonly used to assess the relative importance of stochastic and deterministic assembly processes in community assembly (Sloan et al., 2006; Stegen et al., 2012; Ning et al., 2019). Studies on the development of microbial communities after soil fumigation have shown that deterministic and stochastic assembly processes significantly influence bacterial and fungal communities (Cao et al., 2022).

We studied the long-term effects of CP fumigation on soil microbes in *P. notoginseng* planting field, analyzed the variations in the composition and diversity of bacterial and fungal communities in soil combined with high-throughput gene sequencing, and correlated the changes in the composition of microbial communities with environmental factors like soil physicochemical properties and soil enzyme activities. The NST and niche breadth index describe the differences in the assembly process of microbial communities after CP fumigation for 3 years.

## 2. Materials and methods

### 2.1. Fumigation experiments

We conducted fumigation experiments in The Xin Long Ga Village, Wenshan City, Yunnan Province, China (104°10′8.5 “E, 23°33′25.9 “N) in October 2018. Two treatments in a random block design were assigned as: (1) No fumigation: The soil was not fumigation. (2) CP fumigation: After the soil was plowed and adjusted the absolute water to 60.0% ~ 70.0%, CP (Dalian Lvfeng Chemical Co Ltd., China, 99.5% purity) was injected into the soil at a concentration of 40 g/m^2^ and then immediately covered with 0.04 mm thick polyethylene (PE) film for 30 days, PE film was obtained from Shandong Long Xing Science and Technology Co Ltd. (China).*P. notoginseng* seedlings were transplanted into the fumigated soil after removing the PE film 15 days. *P.notoginseng* were planted 15 cm apart in seedbeds 3.0 cm deep by 1.0 m wide. The row peaks were 50 cm apart. The area of the fumigation treatment plot was 1000.0 m^2^, and no fumigation plot was 100.0 m^2^, and the treatment was triplicated. All the treatments over the 3 years had the same field management practices.

### 2.2. Soil sample collection

The soil was collected in October 2019，2020, and 2021, respectively. In each plot, three points were selected randomly, and soil samples were collected 5.0–20.0 cm below the soil surface. Each sample was divided into three parts. The first part was refrigerated at 4°C for detecting soil pathogens and inorganic nitrogen content; The second part was refrigerated at −80°C for genetic analysis that indicated changes in the soil bacterial and fungal communities; and the third part was air-dried for measurement of changes to the physicochemical properties of soil and the activities of soil enzyme.

### 2.3. *Panax notoginseng* viability rate and plant height

Twenty *P. notoginseng* plants were randomly selected in the treatment plot to judge whether they were alive based on their growth status. The effect of soil fumigation on the viability rate of *P. notoginseng* using the formula Y = (20-X) /20 * 100%, Y represents the survival rate of *P. notoginseng* (%), and X represents the number of dead *P. notoginseng* plants. Plant height indicates the height of the above-ground parts of *P. notoginseng*.

### 2.4. Physicochemical properties of soil

Nitrate nitrogen (NO_3_^−^-N) and ammonium nitrogen (NH_4_^+^-N) were extracted with 2 M KCl and measured by the Futura™ Continuous Flow Analytical System (Alliance Instruments Ltd., France). The P in the soil was extracted with 0.5 M NaHCO_3,_ and its concentration was measured using a UV 2102-P C Spectrophotometer (UNICO, New Jersey, USA) (Olsen, 1954). The available potassium (K) was extracted with 1 M NH_4_COOH. Its concentration was measured using an FP640 Flame Photometer (Shanghai Instruments Group Co., Ltd., Shanghai, China) (Bao, 2010). The content of soil OM was measured by the K_2_Cr_2_O_7_–H_2_SO_4_ oxidation–reduction method (Schinner et al., 2012). The pH values and EC of the soil were measured (soil: water = 1:2.5) using the MP512-02 precision water meter and the MP513 conductivity meter (Shanghai Sanxin Instrument Co., Ltd.), respectively (Bao, 2010), Soil total nitrogen and total carbon were measured by CN 802 carbon and nitrogen analyzer (VELP China Co. LTD, China).

### 2.5. Detection of soil-borne pathogens and soil enzyme activity

The average populations [measured as Colony-Forming Units (CFU) g^−1^ soil] of *Fusarium* spp. and *Phytophthora* spp. in the soil were used to quantify the effect of soil fumigation on soil pathogens. The contents of *Fusarium* spp. and *Phytophthora* spp. in soil were detected according to the methods of Komada (1975) and Masago (1977) respectively. Soil acid phosphatase (S-ACP), neutral soil protease (S-NPT), soil sucrase (S-SC), and soil urease enzyme (S-UE) activities were measured using the corresponding kits (Beijing Solarbio Technology Co., Ltd., China.), respectively. The absorbance of the enzyme was measured using a FlexStation® 3 Multi-Mode Microplate Reader (Molecular Devices LLC., USA), and the enzyme activity was calculated.

### 2.6. DNA extraction, PCR amplification, and high-throughput sequencing

Total soil DNA was extracted following the instructions of the Powersoil®DNA Isolation Kit (Mo Bio Laboratories Inc., USA), the concentration and quality of the extracted DNA were verified using the NanoDrop™ 1,000 (Thermo Fisher Scientific, USA) and 1% agarose gel electrophoresis, respectively (Sun et al., 2022b).

The extracted DNA was used as a template for polymerase chain reaction (PCR) amplification, and MiSeq amplicon sequencing of the 16S rRNA gene V_3_-V_4_ region of soil bacteria and the fungal ITS region, the universal bacterial primer was 338F (ACTCCTACGGGAGGCAGCA)- 806 R (GGACTACHVGGGTWTCTAAT) (Xu et al., 2016) and the universal fungal primer was ITS1F (GGACTACHVGGGTWTCTAAT)-ITS2R (GCTGCGTTCTTCATCGATGC) (Adams et al., 2013).

PCR amplification process was as follows: pre-denaturation at 95°C for 3 min, denaturation at 95°C for 30 s, annealing at 55°C for 30 s, and extension at 72°C for 45 s, followed by a final extension at 72°C for 10 min, for a total of 28 cycles, the PCR products were verified using 2% agarose gel electrophoresis and purified by AxyPrep™ DNA Gel Extraction Kit (Axygen BioSciences Inc., USA) and quantified using a QuantiFluor™-ST fluorometer (Promega Corporation, USA), the purified PCR products were sequenced by Shanghai Meiji Biopharmaceutical Technology Co., Ltd., China.

The soil 16S rRNA gene and fungal ITS gene were amplified using primers 338F (ACTCCTACGGGAGGCAGCA)- 806 R (GGACTACHVGGGTWTCTAAT) (Xu et al., 2016) and ITS1F (GGACTACHVGGGTWTCTAAT)-ITS2R (GCTGCGTTCTTCATCGATGC) (Adams et al., 2013), respectively. Plasmid construction and standard curves were generated following the method described by Fraser et al. (2015). Quantitative PCR was performed on a CFX96 real-time PCR system (Bio-Rad, Hercules, CA, USA) with a total reaction volume of 20 μl. The reaction mixture consisted of 2 μl of soil DNA, 0.4 μl of forward and reverse primers, 10 μl of 2x ChamQ SYBR Color qPCR Master Mix (Nanjing Vazyme Biotech Co., Ltd., China), and 20 μl of ddH_2_O to make up a final volume of 20 μl. The PCR amplification process consisted of an initial denaturation step at 95°C for 5 min, followed by 40 cycles of denaturation at 95°C for 30 s, annealing at 94°C for 30 s, and extension at 72°C for 40 s. The samples were repeated three times.

### 2.7. High-throughput sequencing and bioinformatic analysis

The purified amplicons were pooled in equimolar and paired-end sequenced (2 × 300) on an Illumina MiSeq platform (Illumina, San Diego, USA). The original sequences were sequenced, spliced, quality-controlled, and filtered to obtain optimized sequences (Magoc and Salzberg, 2011), clustered into operable taxonomic units (OTUs) according to similarity levels (Sun et al., 2023).

### 2.8. Statistical analysis

The optimized sequences were compared with the fungal (ITS) Unite Database and the Silva (SSU123) 16S rRNA Database. A confidence threshold of 0.7 was chosen to combine with the RDP classifier Bayesian algorithm v2.2^1^ to taxonomically analyze (Magoc and Salzberg, 2011) OTU sequences at 97% similarity level to obtain species annotation information for OTUs. To analyze the abundance and community structure of bacteria and fungi in the soil, the alpha diversity indices Shannon, Chao, Simpson, and ACE were calculated using the bioinformatics tool Mothur (Sun et al., 2021), and the principal coordinate analysis (PCoA) was performed by the Bray-Curtis algorithm. Linear Discriminant Analysis Effect Size (LEfSe) for analysis of significantly different biomarkers (from phylum to genus) following soil fumigation treatment (*p* > 0.05) (Sun et al., 2022a). We analyzed differences in species abundance distribution between samples based on the unweighted UniFrac distance algorithm. Spearman correlations were calculated between sample OTU abundance and environmental factors, and the result of Spearman visualized using the heatmap package in R (version 3.3.1). Calculation of the NST using the NST package for the R (version 4.0.2) (Ning et al., 2019), the Levins niche breadth index (Levins, 1968; Sun et al., 2020) was calculated using the function niche. Width () from the spaa package of R (version 4.0.2) (Zhang et al., 2018). Statistical analysis was performed using SPSS v19.0 statistical software and Microsoft Excel 2016， a One-Way Analysis of Variance (ANOVA), and Duncan’s Multiple-Range test.

## 3. Results

### 3.1. Changes in soil physicochemical properties and soil enzyme activity

CP fumigation increased soil EC by 18.7% ~ 34.1% (Supplementary Figure S1B). This increased the P content by approximately 34.6 ~ 101.6 mg/kg (Supplementary Figure S1G) and decreased the nitrate (NO_3_^−^-N) content by 10.3% ~ 45.3% (Supplementary Figure S1F). Two years after fumigation, total carbon and nitrogen content in soil were higher in the fumigated plot than in the non-fumigation (Supplementary Figure S1A). In contrast, soil pH was lower in the fumigated plot (Supplementary Figure S1H). The content of K was lower in the early recovery period after than in the non-fumigated plot but significantly higher 3 years after fumigation (Supplementary Figure S1C). There was no significant change in soil NH4^+^-N during the 3 years after fumigation (Supplementary Figure S1E).

**Figure 1:**
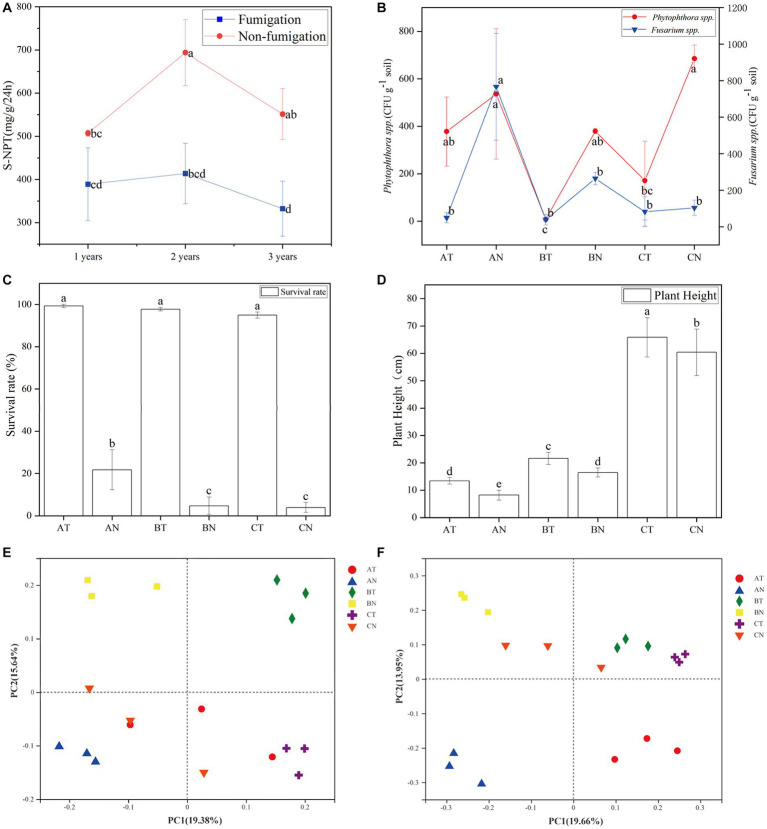
Changes of S-NPT **(A)**, *Fusarium* spp., *Phytophthora* spp. **(B)**, *P. notoginseng*’s survival rate **(C)**, plant height **(D)** and beta diversity **(E,F)** with soil fumigation. According to Duncan’s new Multiple-Range test, means (*N* = 3) within the same period accompanied by the same letter are not statistically different (*p* = 0.05). AT, 1  year after fumigation; AN, 1  year after non-fumigation; BT, 2  years after fumigation; BN, 2  years after non-fumigation; CT, 3  years after fumigation; CN, 3  years after non-fumigation.

CP fumigation significantly decreased the activity of the S-NPT enzyme (Figure 1A). The activity of the S-UE enzyme did not recover to the control level until the second year after fumigation (Supplementary Figure S2B). No significant difference in the activity of the S-ACP and S-SC enzymes after fumigation (Supplementary Figures S1C, S2A).

### 3.2. Changes in soil pathogens and the growth of *Panax notoginseng*

CP fumigation has an inhibitory effect on soil-borne pathogens. The inhibition rate of *Phytophthora* spp. was still 97.8 and 75.0% in the second and third years after fumigation, respectively. The fumigation’s inhibitory impact on *Fusarium* spp. is at least a year-long (Figure 1B). Fumigation with chlorinated enhanced *P. notoginseng’s* survival rate (Figure 1C), stimulated the plant’s growth, and caused a higher plant height in the fumigated plot (Figure 1D).

### 3.3. Changes in the abundance of soil fungi and bacteria

CP fumigation decreased the copy number of the bacterial 16S rRNA gene by 56.4 and 37.7% in the first and second years, respectively, and restored to the control level in the third year as compared to the non-fumigation plot (Supplementary Figure S3A). Three years after, the copy number of soil fungal ITS gene in the fumigation plot was significantly higher than that in the non-fumigation plot (Supplementary Figure S3B). The number of copies of the fungal ITS gene grew yearly with the increase of planting years.

### 3.4. Soil microbial diversity analysis

#### 3.4.1. Alpha diversity

The bacterial Chao1, ACE, Shannon, and Simpson indices recovered to the control levels following 1 year of fumigation. However, the fungal Chao1, ACE, and Shannon indices were lower than the control after 2 years of CP fumigation. Nevertheless, in the third year, all indices except the Simpson index recovered to the control level (Table 1).

**Table 1:** Changes in the diversity index of bacterial and fungal communities.

Microflora	Treatments	Chao1	ACE	Shannon	Simpson
Fungi	AT	334 ± 42 c	339 ± 39 c	2.04 ± 0.26 d	0.2175 ± 0.0506 ab
	AN	543 ± 36 b	527 ± 49 bc	3.09 ± 0.34 b	0.1048 ± 0.0364 bc
	BT	417 ± 36 bc	431 ± 37 bc	1.94 ± 0.14 d	0.3322 ± 0.0588 a
	BN	963 ± 142 a	961 ± 159 a	4.29 ± 0.33 a	0.0403 ± 0.0169 c
	CT	459 ± 29 bc	462 ± 28 bc	2.27 ± 0.23 cd	0.2912 ± 0.0834 a
	CN	559 ± 127 b	558 ± 124 b	2.92 ± 0.37 bc	0.1267 ± 0.0204 bc
Bacteria	AT	2,781 ± 331 b	2,787 ± 335 b	5.86 ± 0.34 a	0.0128 ± 0.0056 a
	AN	3,349 ± 139 ab	3,358 ± 160 ab	6.36 ± 0.08 a	0.0049 ± 0.0004 a
	BT	2,795 ± 184 b	2,774 ± 240 b	5.82 ± 0.26 a	0.0115 ± 0.0044 a
	BN	3,669 ± 185 a	3,662 ± 193 a	6.30 ± 0.33 a	0.0111 ± 0.0093 a
	CT	2,778 ± 119 b	2,768 ± 99 b	5.83 ± 0.25 a	0.0106 ± 0.0047 a
	CN	3,300 ± 589 ab	3,272 ± 581 ab	6.30 ± 0.31 a	0.0063 ± 0.0017 a

AT, 1 year after fumigation; AN, 1 year after non-fumigation; BT, 2 years after fumigation; BN, 2 years after non-fumigation; CT, 3 years after fumigation; CN, 3 years after non-fumigation. The data presented are the means and standard deviations of three separate experiments. Using Duncan’s multiple range test, different letters indicate statistical significance at *p* < 0.05 level.

#### 3.4.2. Beta diversity

Principal coordinate analysis is a systematic clustering method based on the unweighted Unifrac algorithm and beta diversity distance matrix. It intuitively shows the difference between samples according to the cluster distance of samples. According to PCoA analysis, CP fumigation was the primary cause of the variation in soil microbial community composition. In bacterial and fungal communities, PC1 and PC2 contributed 19.66 and 13.95% (Figure 1F), 19.38, and 15.64% (Figure 1E), respectively, to the variation in species composition.

#### 3.4.3. Changes to the phyla of bacterial and fungal communities

**Figure 2:**
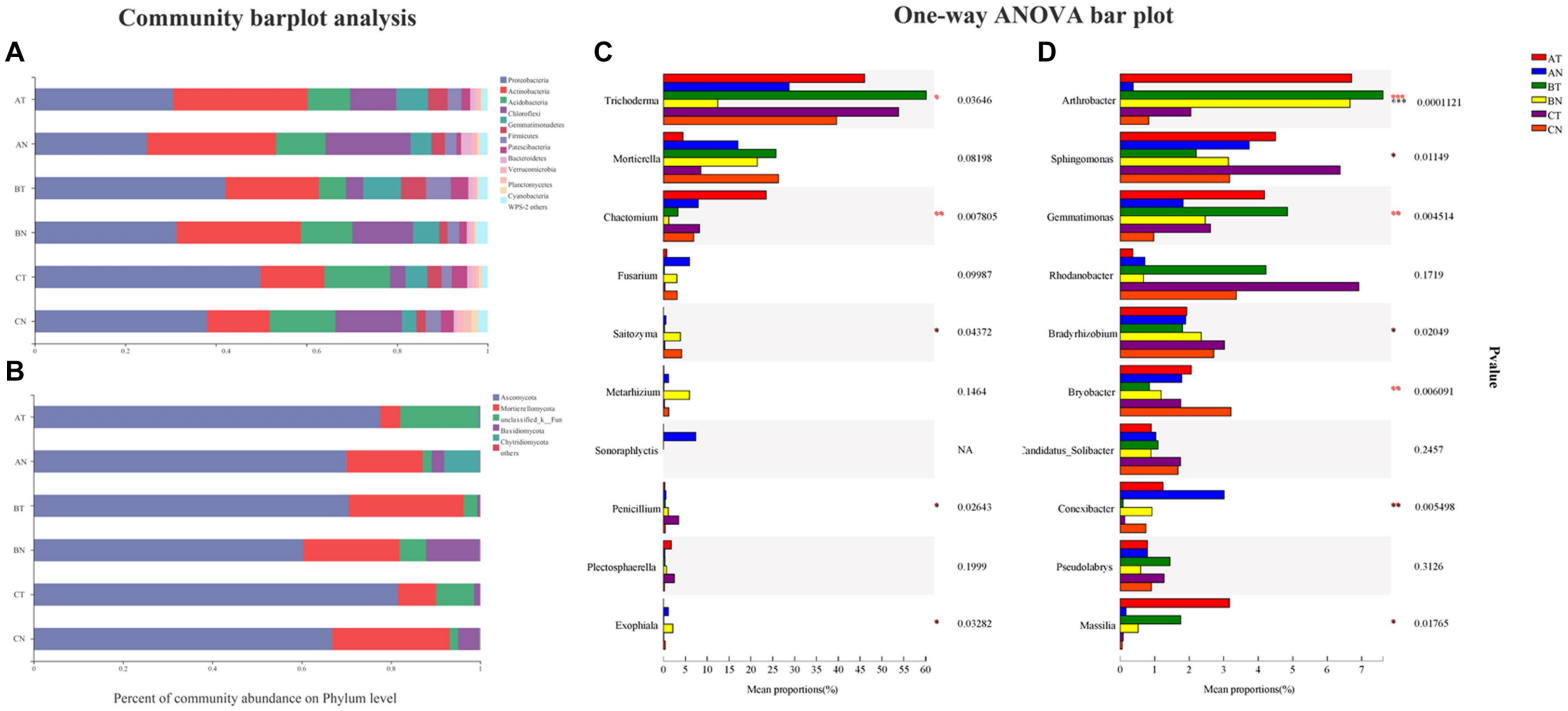
The relative community abundance of bacteria **(A)** and fungi **(B)** at the phylum level. Differences in the relative abundance of fungi **(C)** and bacteria **(D)** and in communities at the genus levels. The number of asterisks indicates significant differences between treatments according to a one-way ANOVA. Tukey–Kramer was used as a *post hoc* test and FDR (False Discovery Rate) adjustment (*p* < 0.05): *0.01 < *p* ≤ 0.05; **0.001 < *p* ≤ 0.01; ***p ≤ 0.001. AT, 1  year after fumigation; AN, 1  year after non-fumigation; BT, 2  years after fumigation; BN, 2  years after non-fumigation; CT, 3  years after fumigation; CN, 3  years after non-fumigation.

At the phylum level, fumigation treatment increased the relative abundance of *Ascomycota* by 10.81% ~ 22.06% and inhibited the quantity of *Basidiomycota* by 71.76% ~ 94.97% (Figure 2B); *Proteobacteria*, *Actinobacteria*, *Acidobacteria*, *Chloroflexi*, and *Gemmatimonadetes* were the dominant species in the bacterial community. CP fumigation increased the relative abundance of *Proteobacteria*, *Gemmatimonadetes,* and *Firmicutes* and inhibited the relative abundance of *Chloroflexi* (Figure 2A).

#### 3.4.4. Changes in the genera composition in the bacterial and fungal communities

*Arthrobacter*, *Sphingomonas*, *Gemmatimonas*, *Rhodanobacter,* and *Bradyrhizobium* were the top five genera of the bacterial community. Compared to non-fumigated plots, fumigation increased the relative abundance of *Arthrobacter Gemmatimonas,* and *Massilia* and decreased the relative abundance of *Conexibacter* by roughly 54.71% ~ 94.2% (Figure 2D). *Trichoderma* was the dominating population in the fungus community. CP fumigation increased *Trichoderma* and *Chaetomium* and decreased the relative abundance of *Fusarium* and *Saitozyma* (Figure 2C).

### 3.5. Differential species analysis of soil bacterial and fungal communities

**Figure 3:**
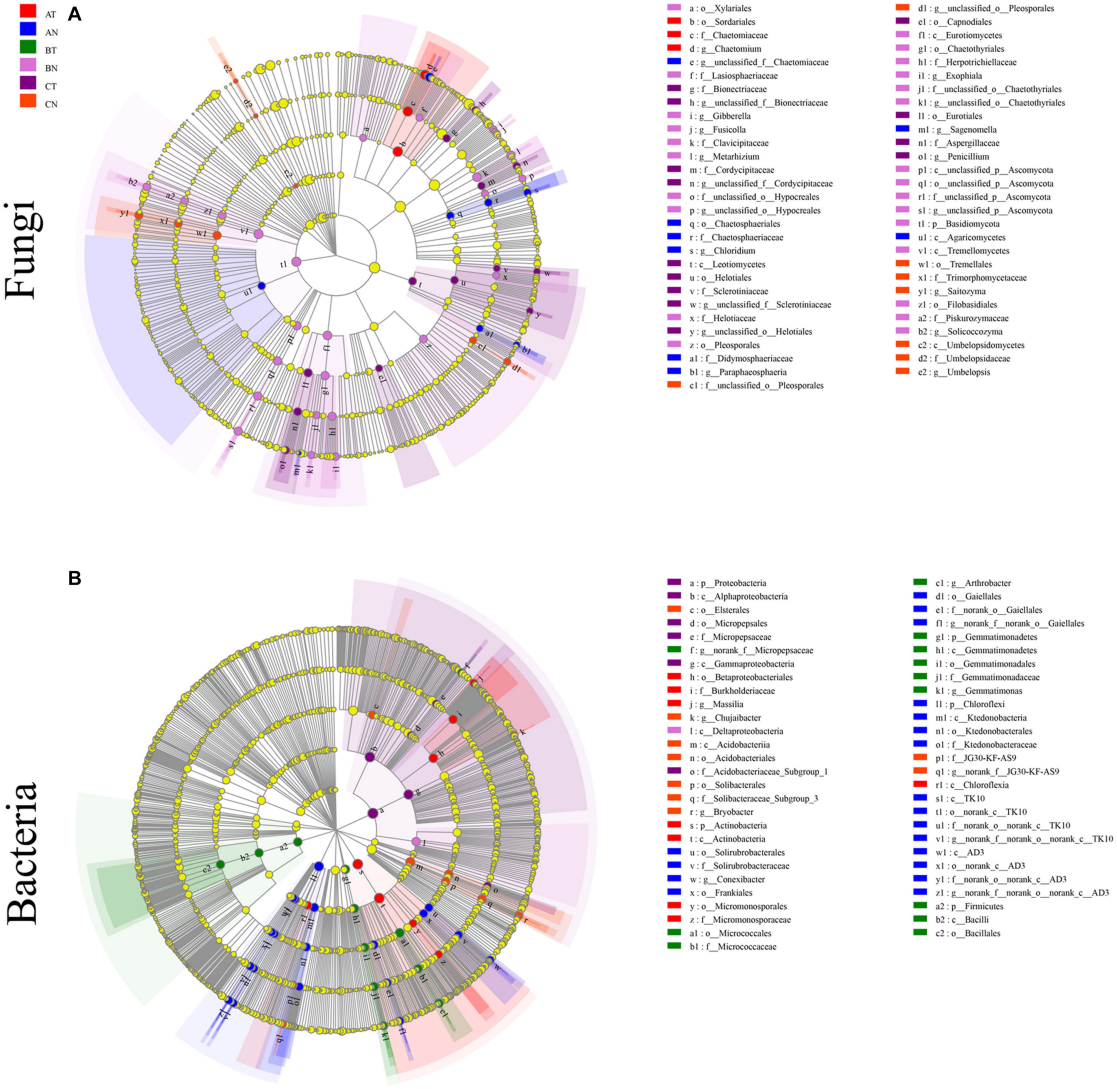
LEfSe cladogram analysis of the differentially abundant species in soil fungal **(A)** and bacterial **(B)** communities (*p* = 0.05, LDA ≥ 3.5). The figure shows five rings in the cladogram, from inside to outside, representing the phylum, class, order, family, and genus, respectively. The different color nodes (except yellow) on the ring represent significant changes in taxonomic composition due to the treatment. AT, 1 year after fumigation; AN, 1  year after non-fumigation; BT, 2  years after fumigation; BN, 2  years after non-fumigation; CT, 3  years after fumigation; CN, 3  years after non-fumigation.

LEfSe analyzed the differences in abundance between fungi and bacteria at the phylum and genus level. At the genus level, LEfSe analysis revealed that the bacterial community had 11 biomarkers (AT, AN, BT, and CN containing 1, 4, 3, and 3, respectively). The fungal community included 21 biomarkers (AT, AN, BN, CT, and CN, each containing 1, 4, 8, and 5 biomarkers, respectively) (Figure 3). The results showed that the bacterial community was more sensitive to CP fumigation than the fungal community.

### 3.6. Correlation analysis of soil properties and microbial communities

**Figure 4:**
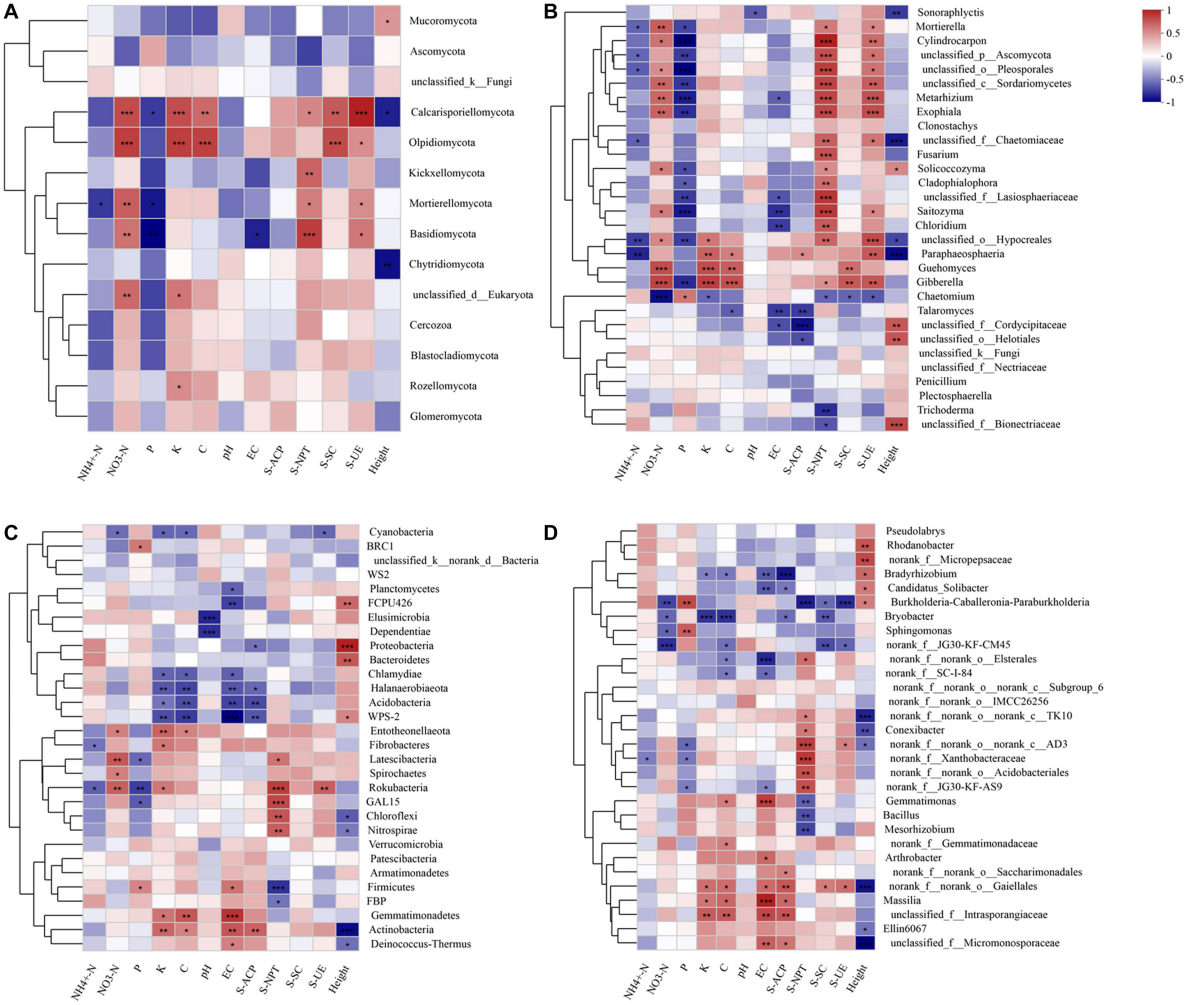
Correlation analysis between microbial community with the soil’s physicochemical properties and enzyme activity at the fungal phylum **(A)** and genus level **(B)**, and the bacterial phylum **(C)** and genus level **(D)**, respectively. The number of asterisks indicates significant differences between treatments according to a one-way ANOVA. Tukey–Kramer was used as a *Post hoc* test and FDR (False Discovery Rate) adjustment (*p* < 0.05): *0.01 < *p* ≤ 0.05; **0.001 < *p* ≤ 0.01; ***p ≤ 0.001.

At the fungal phylum level, *Basidiomycota* positively correlated with NO_3_^−^-N, S-NPT, and S-UE (*R* = 0.59, *R* = 0.77, *R* = 0.58, *p* < 0.05). It displayed a negative correlation with P and EC (*R* = –0.65, *R* = –0.55, *p* < 0.05) (Figure 4A). At the fungal genus level, *Trichoderma* exhibited a negative correlation with S-NPT (*R* = –0.65, *p* < 0.05); Fusarium displayed a positive correlation with S-NPT (*R* = 0.71, *p* < 0.05); *Saitozyma* showed a positive correlation with NO_3_^−^-N, S-NPT, and S-UE (*R* = 0.55, *R* = 0.80, *R* = 0.50, *p* < 0.05), and a negative correlation with P and EC (*R* = –0.72, *R* = –0.66, *p* < 0.05) (Figure 4B).

At the bacterial phylum level, *Proteobacteria* exhibited a negative correlation with S-ACP (*R* = –0.50, *p* < 0.05); *Actinobacteria* showed a positive correlation with K, C, and EC (*R* = 0.60, *R* = 0.49, *R* = 0.66, *p* < 0.05), while *Acidobacteria* displayed a negative correlation with K, C, EC, and S-ACP (*R* = –0.49, *R* = –0.64, *R* = –0.66, *R* = –0.64, *p* < 0.05). *Chloroflexi* demonstrated a positive correlation with S-NPT (*R* = 0.67, *p* < 0.05); *Gemmatimonadetes* exhibited a positive correlation with K, C, and EC (*R* = 0.54, *R* = 0.66, *R* = 0.80, *p* < 0.05) (Figure 4C). At the bacterial genus level: *Arthrobacter* exhibited a positive correlation with EC (*R* = 0.58, *p* < 0.05); *Sphingomonas* displayed a negative correlation with NO_3_^−^-N (*R* = –0.58, *p* < 0.05) and a positive correlation with P (*R* = 0.62, *p* < 0.05). *Gemmatimonas* showed a positive correlation with C and EC (*R* = 0.49, *R* = 0.75, *p* < 0.05) and a negative correlation with S-NPT (*R* = –0.6, *p* < 0.05). *Bradyrhizobium* exhibited a negative correlation with K, C, EC, and S-ACP (*R* = –0.47, *R* = –0.51, *R* = –0.61, *R* = –0.85, *p* < 0.05) (Figure 4D).

### 3.7. The impact on the deterministic and stochastic processes involved in microbial community assembly

**Figure 5:**
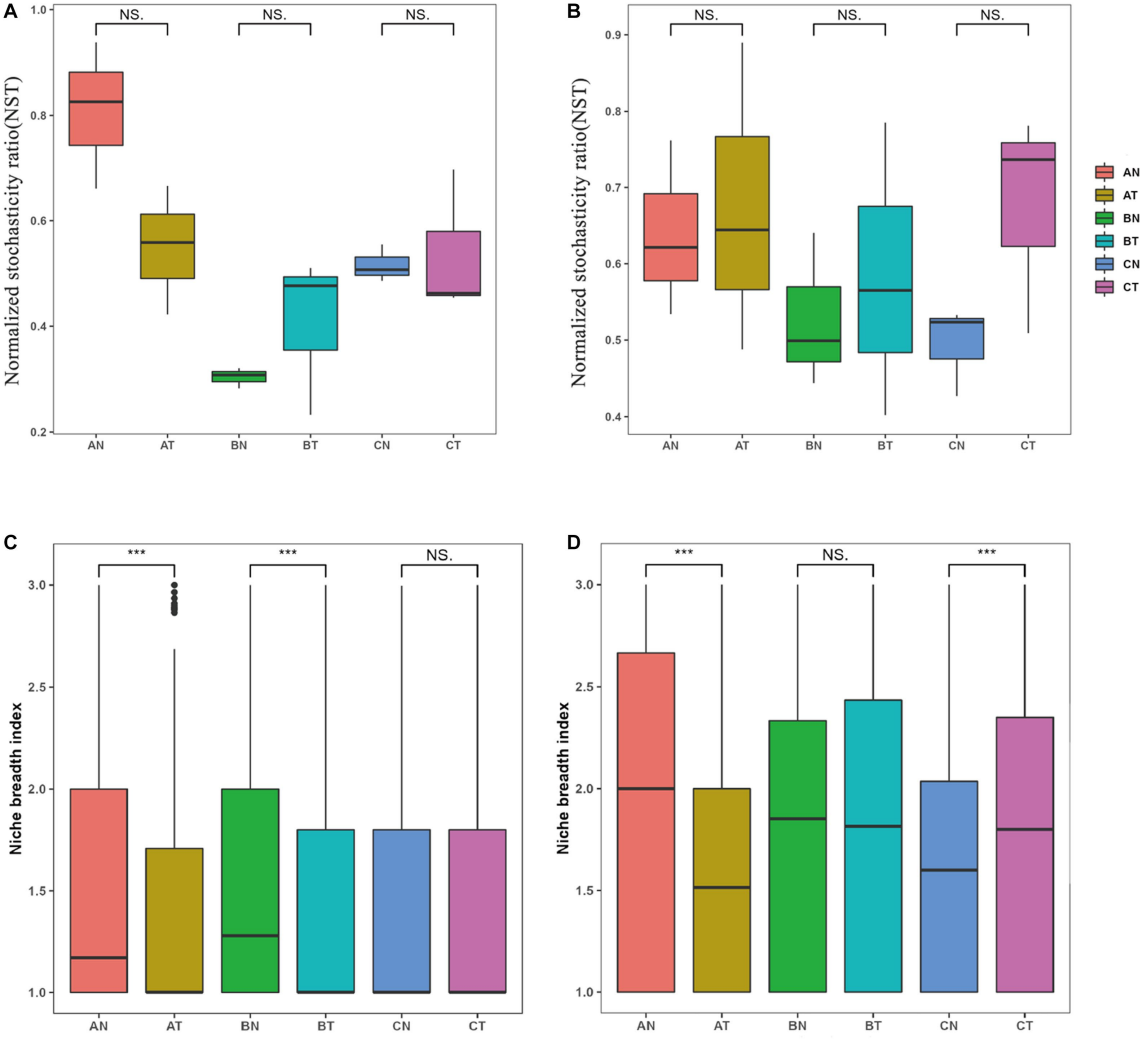
Effects of fumigation on Normalized stochasticity ratio (NST) of fungi **(A)** and bacteria **(B)**, niche breadth of fungi **(C)** and bacteria **(D)**. The number of asterisks indicates significant differences between treatments according to a one-way ANOVA. (*p* < 0.05): *0.01 < *p* ≤ 0.05; **0.001 < *p* ≤ 0.01; ***p ≤ 0.001; NS representative of *p* > 0.05; AT, 1  year after fumigation; AN, 1  year after non-fumigation; BT, 2  years after fumigation; BN, 2  years after non-fumigation; CT, 3  years after fumigation; CN, 3  years after non-fumigation.

The NST evaluated the relative importance of stochastic and deterministic assembly processes in the soil microbial community. During the assembly process of soil microbial communities after CP fumigation, the NST values of bacteria were all more than 50%, indicating that stochastic processes dominated the assembly of bacterial communities (Figure 5B). Compared with the control group, fumigation treatment increased the dominance of stochastic processes in bacterial community assembly by 5.44–36.64%, indicating that fumigation enhanced the role of stochasticity in the assembly of bacterial communities. For the assembly process of fungal communities, stochasticity dominated in the early stage of microbial recovery, that is, 2 years after fumigation. At the same time, deterministic methods were dominant in the later stage of recovery, that is, the third year after fumigation (Figure 5A).

The soil fumigation narrowed the niche breadth of the fungus, which returned to pre-fumigation levels after 3 years (Figure 5C). The bacteria’s niche breadth was likewise decreased by soil fumigation. Still, as recovery time increased, it gradually widened and was significantly higher in the third year compared to the unfumigated treatment (Figure 5D). By comparing the average niche breadth based on the OUT level, the niche breadth of bacteria was higher than that of fungi, indicating that bacteria have a more elevated ecological niche.

## 4. Discussion

### 4.1. Physicochemical properties of soil and *Panax notoginseng* viability rate

We found that the P in *P. notoginseng* continuous cropping soil showed an increasing trend in contrast to previous records (Zhang Y. et al., 2019). The result might be related to the water and fertilizer management in the field during *P. notoginseng* growth. As the consecutive cropping years increase, the degree of soil salinization worsens (Libutti et al., 2018), providing a more suitable environment for breeding soil pathogens (Shrivastava and Kumar, 2015). This increases the incidence rate of soil-borne diseases in *P. notoginseng* and leads to a gradual decline in its survival rate yearly. The composition of the rhizosphere microbial community in *P. notoginseng* is closely related to its continuous cropping obstacles (Tan et al., 2017). CP fumigation reduces the abundance of *Phytophthora* and *Fusarium* in the soil and the incidence of soil-borne diseases, improves the survival rate of *P. notoginseng*, and is consistent with the research results of previous studies (Zhang et al., 2019). This may be related to breaking continuous cropping obstacles caused by the accumulation of soil-borne pathogens after CP fumigation treatment.

There is a positive correlation between the abundance of *Fusarium* and neutral protease activity, and *Fusarium oxysporum* has been reported to produce neutral protease (Bourosh et al., 2013). Reports showed that protease activity exhibits the same trend as NO_3_^−^-N content (Zhao et al., 2022). After CP fumigation treatment, we found that both soil-neutral protease activity and NO_3_^−^-N content decreased, related to the transformation of protease and nitrogen (Yuan et al., 2017).

### 4.2. The impact of soil fumigation on soil microorganism

CP fumigation increased the relative abundance of *Ascomycota*. *Trichoderma* and *Chaetomium*, which are members of the *Ascomycota,* also increased. *Trichoderma*, as a kind of biocontrol fungus (Stoppacher et al., 2010), plays an essential role in preventing and controlling plant diseases (Heye and Andrews, 1983). It is reported that *Trichoderma* in the rhizosphere can enhance the systemic immunity of plants, which may also be one of the factors contributing to the increased survival rate of *P. notoginseng* (Harman, 2000). The fungal endophyte *Chaetomium globosum* was isolated from the medicinal plant *Ginkgo biloba*. Its crude fungal fermentation extract can inhibit the phytopathogenic fungi *Rhizopus stolonifer* and *Coniothyrium diplodiella* (Zhang et al., 2013). The relative abundance of *Fusarium* in the soil decreased after fumigation, which is consistent with the results of previous studies (Zhang et al., 2020), probably because *Chaetomium* can produce secondary metabolites that can inhibit soil-borne pathogens.

*Proteobacteria* is the most abundant phylum of bacteria in soil (Janssen, 2006) and can be easily found in nutrient-rich environments (Taketani et al., 2013). Soil fumigation with CP increased the relative abundance of *Proteobacteria*, which contradicts the findings of previous studies (Zhang et al., 2019) and may be related to the different crops planted after fumigation. Fumigation reduced the relative abundance of *Chloroflexi* and increased that of *Gemmatimonadetes,* consistent with the results of previous studies (Fang et al., 2019). *Chloroflexi* has a potential role in carbon cycling (Islam et al., 2019) and is involved in respiration, fermentation, carbon dioxide fixation, and substrate-level phosphorylation, including phosphorylation sugars (Hug, 2013).

The fungal diversity did not return to the control level 3 years after fumigation, but the variety of the bacteria had recovered. The results showed that the microbial diversity could be restored after fumigation, and the recovery rate differed. It is reported that after fumigation, aided by the organic fertilizer, microbial fertilizer, and humic acid can promote the recovery of microbial diversity (Cheng et al., 2021; Li Q. et al., 2022; Pu et al., 2022).

### 4.3. Effect of soil fumigation on microbial community assembly

The NST characterizes the importance of the deterministic and stochastic processes involved in microbial community assembly (Cheng et al., 2021). We found that stochasticity dominates the assembly process of bacterial communities after fumigation. In contrast, the early stage of fungal communities is mainly driven by stochasticity, while deterministic processes dominate the later stage. Following the injection of organic carbon into groundwater environments, Ning et al. observed a transition in the microbial assembly process from deterministic processes to stochasticity, with deterministic processes regaining dominance in the later stages as organic carbon was depleted (Ning et al., 2019). We found that the K in the soil decreased in the early stage and increased in the later stage after fumigation. It has been reported that soil microbial participate in solubilizing insoluble and fixed forms of K (Zarjani et al., 2013; Raghavendra et al., 2016).

*Aspergillus terreus* and *Aspergillus niger* have been reported to be associated with the formation of available K (Chang, 2023), and fumigation resulted in a decrease in the relative abundance of *Aspergillus*. However, with increasing recovery time, the relative abundance of *Aspergillus* recovered to the pre-fumigation levels. Therefore, changes in available K content in the soil may be one of the factors influencing the assembly of fungal communities.

### 4.4. The effect of fumigation on microbial ecological niche

Niche theory is a fundamental concept for explaining species coexistence and competition in natural communities (Leibold, 1995), which reveals the degree and capacity of resource utilization, as well as the range and relative position of occupying ecological space within the community (Vergnon et al., 2009; Turnbull et al., 2016).

CP fumigation reduced the niche breadth of fungi and bacteria, indicating that fumigation intensifies microbial resource competition, resulting in narrower ecological niches for bacteria and fungi. When comparing the mean niche breadth based on OTU level, bacteria have a higher niche breadth than fungi. Reports show that narrower niche breadth is more influenced by environmental determinism. In contrast, broader ecological niches are mainly influenced by stochastic (Pandit et al., 2009), which can better explain the community assembly process of bacteria and fungi in this experiment.

## Data availability statement

The datasets presented in this study can be found in online repositories. The names of the repository/repositories and accession number(s) can be found below: NCBI – PRJNA979806.

## Author contributions

XW: methodology and writing – original draft. QW and WL: methodology, software, and investigation. DZ and WF: visualization and investigation. YL and QXW: writing review and editing. AC: validation. DY: conceptualization and supervision. All authors contributed to the article and approved the submitted version.

## Funding

We are grateful for the financial support from the National Natural Science Foundation Project of China (32172462) National Key Research and Development Program of China (2022YFC3501502) over the years.

## Conflict of interest

The authors declare that the research was conducted in the absence of any commercial or financial relationships that could be construed as a potential conflict of interest.

## Publisher’s note

All claims expressed in this article are solely those of the authors and do not necessarily represent those of their affiliated organizations, or those of the publisher, the editors and the reviewers. Any product that may be evaluated in this article, or claim that may be made by its manufacturer, is not guaranteed or endorsed by the publisher.

## Footnotes

^1^http://sourceforge.net/projects/rdp-classifier/

## Supplementary material

The Supplementary material for this article can be found online at:

https://www.frontiersin.org/articles/10.3389/fmicb.2023.1225944/full#supplementary-material

## References

[ref1] Adams R. I. Miletto M. Taylor J. W. Bruns T. D. (2013). Dispersal in microbes: fungi in indoor air are dominated by outdoor air and show dispersal limitation at short distances. ISME J. 7, 1262–1273. doi: 10.1038/ismej.2013.28 23426013 PMC3695294

[ref2] Bao S. D. (2010). Soil agrochemical analysis. third Edn China Agricultural Press, 103–109.

[ref3] Bourosh P. N. Coropceanu E. B. Ciloci A. A. Clapco S. F. Bologa O. A. Bivol C. M.. (2013). New co (III) dioximates with hexafluorophosphate ion as stimulators of the proteolytic activity of the micromycete *Fusarium gibbosum* CNMN FD 12. Russ. J. Coord. Chem. 39, 777–786. doi: 10.1134/S107032841311002X

[ref8] Cao A. Fang W. Li Y. Yan D. Wang Q. Guo M.. (2022). Review on 60 years of soil fumigation and disinfestation in China. Journal of Plant Protection 49, 325–335. doi: 10.13802/j.cnki.zwbhxb.2022.2022822

[ref4] Chang P.-K. (2023). A simple CRISPR/Cas9 system for efficiently targeting genes of *Aspergillus* section Flavi species, *Aspergillus nidulans*, *Aspergillus fumigatus*, *Aspergillus terreus*, and *Aspergillus niger*, microbiology. Sectrum 11, e04648–e04622. doi: 10.1128/spectrum.04648-22 PMC9927283 36651760

[ref5] Cheng H. Zhang D. Ren L. Song Z. Li Q. Jiajia W.. (2021). Bio-activation of soil with beneficial microbes after soil fumigation reduces soil-borne pathogens and increases tomato yield. Environ. Pollut. 283:117160. doi: 10.1016/j.envpol.2021.117160 33878684

[ref6] Dangi S. R. Gerik J. S. Tirado-Corbalá R. Ajwa H. (2015). Soil microbial community structure and target organisms under different fumigation treatments. Appl. Environ. Soil Sci. 2015, 1–8. doi: 10.1155/2015/673264

[ref7] Fan Z. Y. Miao C. P. Qiao X. G. Zheng Y. K. Chen H. H. Chen Y. W.. (2016). Diversity, distribution, and antagonistic activities of rhizobacteria of *Panax notoginseng*. J. Ginseng Res. 40, 97–104. doi: 10.1016/j.jgr.2015.05.003, PMID: 27158229 PMC4845043

[ref9] Fang W. Wang X. Huang B. Zhang D. Liu J. Zhu J.. (2020). Comparative analysis of the effects of five soil fumigants on the abundance of denitrifying microbes and changes in bacterial community composition. Ecotoxicol. Environ. Saf. 187:109850. doi: 10.1016/j.ecoenv.2019.109850, PMID: 31677569

[ref10] Fang W. Yan D. Wang Q. Huang B. Ren Z. Wang X.. (2019). Changes in the abundance and community composition of different nitrogen cycling groups in response to fumigation with 1,3-dichloropropene. Sci. Total Environ. 650, 44–55. doi: 10.1016/j.scitotenv.2018.08.432 30196225

[ref11] Fraser T. Lynch D. H. Entz M. H. Dunfield K. E. (2015). Linking alkaline phosphatase activity with bacterial phoD gene abundance in soil from a long-term management trial. Geoderma 257, 115–122. doi: 10.1016/j.geoderma.2014.10.016

[ref12] Guo R. J. Liu X. Z. Li S. D. Miao Z. Q. (2009). In vitro inhibition of fungal root-rot pathogens of *Panax notoginseng* by Rhizobacteria. Plant Pathol. J. 25, 70–76. doi: 10.5423/PPJ.2009.25.1.070

[ref13] Harman G. E. (2000). Myths and dogmas of biocontrol changes in perceptions derived from research on *Trichoderma harzinum* T-22. Plant Dis. 84, 377–393. doi: 10.1094/PDIS.2000.84.4.377, PMID: 30841158

[ref14] Heye C. C. Andrews J. H. (1983). Antagonism of *Athelia bombacina* and *Chaetomium globosum* to the apple scab pathogen, *Venturia inaequalis*. Phytopathology 73, 650–654. doi: 10.1094/Phyto-73-650

[ref15] Hug L. A. (2013). Community genomic analyses constrain the distribution of metabolic traits across the *Chloroflexi phylum* and indicate roles in sediment carbon cycling. Microbiome 1:22. doi: 10.1186/2049-2618-1-22, PMID: 24450983 PMC3971608

[ref16] Islam Z. F. Cordero P. R. F. Feng J. Chen Y. J. Bay S. K. Jirapanjawat T.. (2019). Two Chloroflexi classes independently evolved the ability to persist on atmospheric hydrogen and carbon monoxide. ISME J. 13, 1801–1813. doi: 10.1038/s41396-019-0393-0, PMID: 30872805 PMC6776052

[ref17] Janssen P. H. (2006). Identifying the dominant soil bacterial taxa in libraries of 16S rRNA and 16S rRNA genes. Appl. Environ. Microbiol. 72, 1719–1728. doi: 10.1128/AEM.72.3.1719-1728.2006, PMID: 16517615 PMC1393246

[ref18] Komada H. (1975). Development of a selective medium for quantitative isolation of *Fusarium oxysporum* from natural soil. Rev. Plant Protect. Res. 8, 114–124.

[ref19] Leibold M. A. (1995). The niche concept revisited-mechanistic models and community context. Ecology 76, 1371–1382. doi: 10.2307/1938141

[ref20] Levins R. (1968). Evolution in changing environments: some theoretical explorations. Princeton: Princeton University Press.

[ref21] Li J. Chen Y. Qin X. Cao A. Lu A. (2022). Impact of biochar on rhizosphere bacterial diversity restoration following chloropicrin fumigation of planted soil. Int. J. Environ. Res. Public Health 19:2126. doi: 10.3390/ijerph19042126 35206314 PMC8872450

[ref22] Li H. Deng C. Q. Chen B. Y. Zhang S. P. Liang Y. Luo X. G. (2009). Total saponins of *Panax notoginseng* modulate the expression of caspases and attenuate apoptosis in rats following focal cerebral ischemia-reperfusion. J. Ethnopharmacol. 121, 412–418. doi: 10.1016/j.jep.2008.10.042, PMID: 19059471

[ref23] Li Q. Zhang D. Cheng H. Ren L. Jin X. Fang W.. (2022). Organic fertilizers activate soil enzyme activities and promote the recovery of soil beneficial microorganisms after dazomet fumigation. J. Environ. Manag. 309:114666. doi: 10.1016/j.jenvman.2022.114666, PMID: 35151999

[ref24] Libutti A. Cammerino A. R. B. Monteleone M. (2018). Risk assessment of soil salinization due to tomato cultivation in Mediterranean climate conditions. Water 10:1503. doi: 10.3390/w10111503

[ref25] Ma L. Cao Y. H. Cheng M. H. Huang Y. Mo M. H. Wang Y.. (2013). Phylogenetic diversity of bacterial endophytes of Panax notoginseng with antagonistic characteristics towards pathogens of root-rot disease complex. Antonie Van Leeuwenhoek 103, 299–312. doi: 10.1007/s10482-012-9810-3 22987248

[ref26] Magoc T. Salzberg S. L. (2011). FLASH: fast length adjustment of short reads to improve genome assemblies. Bioinformatics 27, 2957–2963. doi: 10.1093/bioinformatics/btr507, PMID: 21903629 PMC3198573

[ref27] Masago H. (1977). Selective inhibition of Pythium spp. on a medium for direct isolation of *Phytophthora* spp. from soils and plants. Phytopathology 67, 425–428.

[ref28] Ning D. Deng Y. Tiedje J. M. Zhou J. (2019). A general framework for quantitatively assessing ecological stochasticity. Proc. Natl. Acad. Sci. U. S. A. 116, 16892–16898. doi: 10.1073/pnas.1904623116, PMID: 31391302 PMC6708315

[ref29] Olsen S. R. (1954). Estimation of available phosphorus in soils by extraction with sodium bicarbonate. Washington: US Department of Agriculture.

[ref30] Pandit S. N. Kolasa J. Cottenie K. (2009). Contrasts between habitat generalists and specialists: an empirical extension to the basic metacommunity framework. Ecology 90, 2253–2262. doi: 10.1890/08-0851.1 19739387

[ref31] Pu R. Wang P. Guo L. Li M. Cui X. Wang C.. (2022). The remediation effects of microbial organic fertilizer on soil microorganisms after chloropicrin fumigation. Ecotoxicol. Environ. Saf. 231:113188. doi: 10.1016/j.ecoenv.2022.113188, PMID: 35051756

[ref32] Raghavendra M P Chandra Nayaka S Nuthan B R. (2016). Role of rhizosphere microflora in potassium solubilization. Potassium solubilizing microorganisms for sustainable agriculture. 43–59. doi: 10.1007/978-81-322-2776-2_4

[ref33] Rashid M. I. Mujawar L. H. Shahzad T. Almeelbi T. Ismail I. M. Oves M. (2016). Bacteria and fungi can contribute to nutrients bioavailability and aggregate formation in degraded soils. Microbiol. Res. 183, 26–41. doi: 10.1016/j.micres.2015.11.007 26805616

[ref1001] Rodríguez A. Castrejón-Godínez M. L. Salazar-Bustamante E. Gama-Martínez Y. Sánchez-Salinas P. Mussali-Galante E.. (2020). Omics approaches to pesticide biodegradation. Current Microbiology 77, 545–563. doi: 10.1007/s00284-020-01916-5 32078006

[ref34] Santos-Medellin C. Liechty Z. Edwards J. Nguyen B. Huang B. Weimer B. C.. (2021). Prolonged drought imparts lasting compositional changes to the rice root microbiome. Nat Plants 7, 1065–1077. doi: 10.1038/s41477-021-00967-1 34294907

[ref35] Schinner F. Öhlinger R. Kandeler E. Margesin R. (2012). Methods in soil biology. Berlin: Springer Science & Business Media.

[ref36] Shrivastava P. Kumar R. (2015). Soil salinity: a serious environmental issue and plant growth promoting bacteria as one of the tools for its alleviation. Saudi J Biol Sci 22, 123–131. doi: 10.1016/j.sjbs.2014.12.001, PMID: 25737642 PMC4336437

[ref37] Sloan W. T. Lunn M. Woodcock S. Head I. M. Nee S. Curtis T. P. (2006). Quantifying the roles of immigration and chance in shaping prokaryote community structure. Environ. Microbiol. 8, 732–740. doi: 10.1111/j.1462-2920.2005.00956.x, PMID: 16584484

[ref38] Stegen J. C. Lin X. Konopka A. E. Fredrickson J. K. (2012). Stochastic and deterministic assembly processes in subsurface microbial communities. ISME J. 6, 1653–1664. doi: 10.1038/ismej.2012.22, PMID: 22456445 PMC3498916

[ref39] Stoppacher N. Kluger B. Zeilinger S. Krska R. Schuhmacher R. (2010). Identification and profiling of volatile metabolites of the biocontrol fungus *Trichoderma atroviride* by HS-SPME-GC-MS. J. Microbiol. Methods 81, 187–193. doi: 10.1016/j.mimet.2010.03.011, PMID: 20302890

[ref40] Sun R. Chen Y. Han W. Dong W. Zhang Y. Chunsheng H.. (2020). Different contribution of species sorting and exogenous species immigration from manure to soil fungal diversity and community assemblage under long-term fertilization. Soil Biol. Biochem. 151:108049. doi: 10.1016/j.soilbio.2020.108049

[ref41] Sun R. Ding J. Li H. Wang X. Li W. Li K.. (2023). Mitigating nitrate leaching in cropland by enhancing microbial nitrate transformation through the addition of liquid biogas slurry, agriculture. Ecosyst Environ 345:108324. doi: 10.1016/j.agee.2022.108324

[ref42] Sun R. Niu J. Luo B. Wang X. Li W. Zhang W.. (2022a). Substitution of manure for mineral P fertilizers increases P availability by enhancing microbial potential for organic P mineralization in greenhouse soil. Front. Bioeng. Biotechnol. 10:1078626. doi: 10.3389/fbioe.2022.1078626, PMID: 36561049 PMC9763603

[ref43] Sun R. Wang F. Chunsheng H. Liu B. (2021). Metagenomics reveals taxon-specific responses of the nitrogen-cycling microbial community to long-term nitrogen fertilization. Soil Biol. Biochem. 156:108214. doi: 10.1016/j.soilbio.2021.108214

[ref44] Sun R. Zhang W. Liu Y. Yun W. Luo B. Chai R.. (2022b). Changes in phosphorus mobilization and community assembly of bacterial and fungal communities in rice rhizosphere under phosphate deficiency. Front. Microbiol. 13:953340. doi: 10.3389/fmicb.2022.953340 35992700 PMC9382406

[ref45] Taketani R. G. Lima A. B. da Conceicao Jesus E. Teixeira W. G. Tiedje J. M. Tsai S. M. (2013). Bacterial community composition of anthropogenic biochar and Amazonian anthrosols assessed by 16S rRNA gene 454 pyrosequencing. Antonie Van Leeuwenhoek 104, 233–242. doi: 10.1007/s10482-013-9942-0, PMID: 23743632

[ref46] Tan Y. Cui Y. Li H. Kuang A. Li X. Wei Y.. (2017). Rhizospheric soil and root endogenous fungal diversity and composition in response to continuous *Panax notoginseng* cropping practices. Microbiol. Res. 194, 10–19. doi: 10.1016/j.micres.2016.09.009, PMID: 27938858

[ref1002] Tebo B. M. Johnson H. A. McCarthy J. K. Templeton A. S. (2005). Geomicrobiology of manganese(II) oxidation. Trends Microbiol. 13, 421–8. doi: 10.1016/j.tim.2005.07.009 16054815

[ref47] Turnbull L. A. Isbell F. Purves D. W. Loreau M. Hector A. (2016). Understanding the value of plant diversity for ecosystem functioning through niche theory. Proc. Biol. Sci. 283:20160536. doi: 10.1098/rspb.2016.0536 27928043 PMC5204137

[ref48] Vergnon R. Dulvy N. K. Freckleton R. P. (2009). Niches versus neutrality: uncovering the drivers of diversity in a species-rich community. Ecol. Lett. 12, 1079–1090. doi: 10.1111/j.1461-0248.2009.01364.x 19747181

[ref49] Xu N. Tan G. Wang H. Gai X. (2016). Effect of biochar additions to soil on nitrogen leaching, microbial biomass and bacterial community structure. Eur. J. Soil Biol. 74, 1–8. doi: 10.1016/j.ejsobi.2016.02.004

[ref50] Yan D. Wang Q. Song Z. Fang W. Wang Q. Li Y.. (2022). Activation effect of soil available nitrogen, manganese and cobalt after addition of different fumigants, environmental research. Communications 4:041002. doi: 10.1088/2515-7620/ac64ed

[ref51] Yuan X. Wen A. Desta S. T. Dong Z. Shao T. (2017). Effects of four short-chain fatty acids or salts on the dynamics of nitrogen transformations and intrinsic protease activity of alfalfa silage. J. Sci. Food Agric. 97, 2759–2766. doi: 10.1002/jsfa.8103 27754550

[ref52] Zarjani K. Javad N. A. Oustan S. Emadi M. Ahmadi A. (2013). Isolation and characterization of potassium solubilizing bacteria in some Iranian soils. Arch. Agron. Soil Sci. 59, 1713–1723. doi: 10.1080/03650340.2012.756977

[ref53] Zhang D. Yan D. Cheng H. Fang W. Huang B. Wang X.. (2020). Effects of multi-year biofumigation on soil bacterial and fungal communities and strawberry yield. Environ. Pollut. 256:113415. doi: 10.1016/j.envpol.2019.113415, PMID: 31672346

[ref54] Zhang D. Yan D. Fang W. Huang B. Wang X. Wang X.. (2019). Chloropicrin alternated with biofumigation increases crop yield and modifies soil bacterial and fungal communities in strawberry production. Sci. Total Environ. 675, 615–622. doi: 10.1016/j.scitotenv.2019.04.222, PMID: 31035200

[ref55] Zhang J. Zhang B. Liu Y. Guo Y. Shi P. Wei G. (2018). Distinct large-scale biogeographic patterns of fungal communities in bulk soil and soybean rhizosphere in China. Sci. Total Environ. 644, 791–800. doi: 10.1016/j.scitotenv.2018.07.016 29990927

[ref56] Zhang G. Zhang Y. Qin J. Qu X. Liu J. Li X.. (2013). Antifungal metabolites produced by *Chaetomium globosum* no.04, an endophytic fungus isolated from *Ginkgo biloba*. Indian J. Microbiol. 53, 175–180. doi: 10.1007/s12088-013-0362-7, PMID: 24426105 PMC3626953

[ref57] Zhang Y. Zheng Y. Xia P. Xun L. Liang Z. (2019). Impact of continuous *Panax notoginseng* plantation on soil microbial and biochemical properties. Sci. Rep. 9:13205. doi: 10.1038/s41598-019-49625-9, PMID: 31519939 PMC6744506

[ref58] Zhao L. Li Y. Ren W. Huang Y. Wang X. Fu Z.. (2020). Pesticide residues in soils planted with *Panax notoginseng* in South China, and their relationships in *Panax notoginseng* and soil. Ecotoxicol. Environ. Saf. 201:110783. doi: 10.1016/j.ecoenv.2020.110783, PMID: 32534333

[ref59] Zhao Y. Wang Y. Sun S. Liu W. Zhu L. Yan X. (2022). Different forms and proportions of exogenous nitrogen promote the growth of alfalfa by increasing soil enzyme activity. Plan. Theory 11:1057. doi: 10.3390/plants11081057, PMID: 35448784 PMC9029003

