# Supplementary material for: Long-term effects of chloropicrin fumigation on soil microbe recovery and growth promotion of Panax notoginseng
Source: Front Microbiol. 2023 Jul 14;14:1225944. doi: 10.3389/fmicb.2023.1225944 (PMC10375714; doi:10.3389/fmicb.2023.1225944)
Supplement: Supplementary file 1 [file Data_Sheet_1.docx]

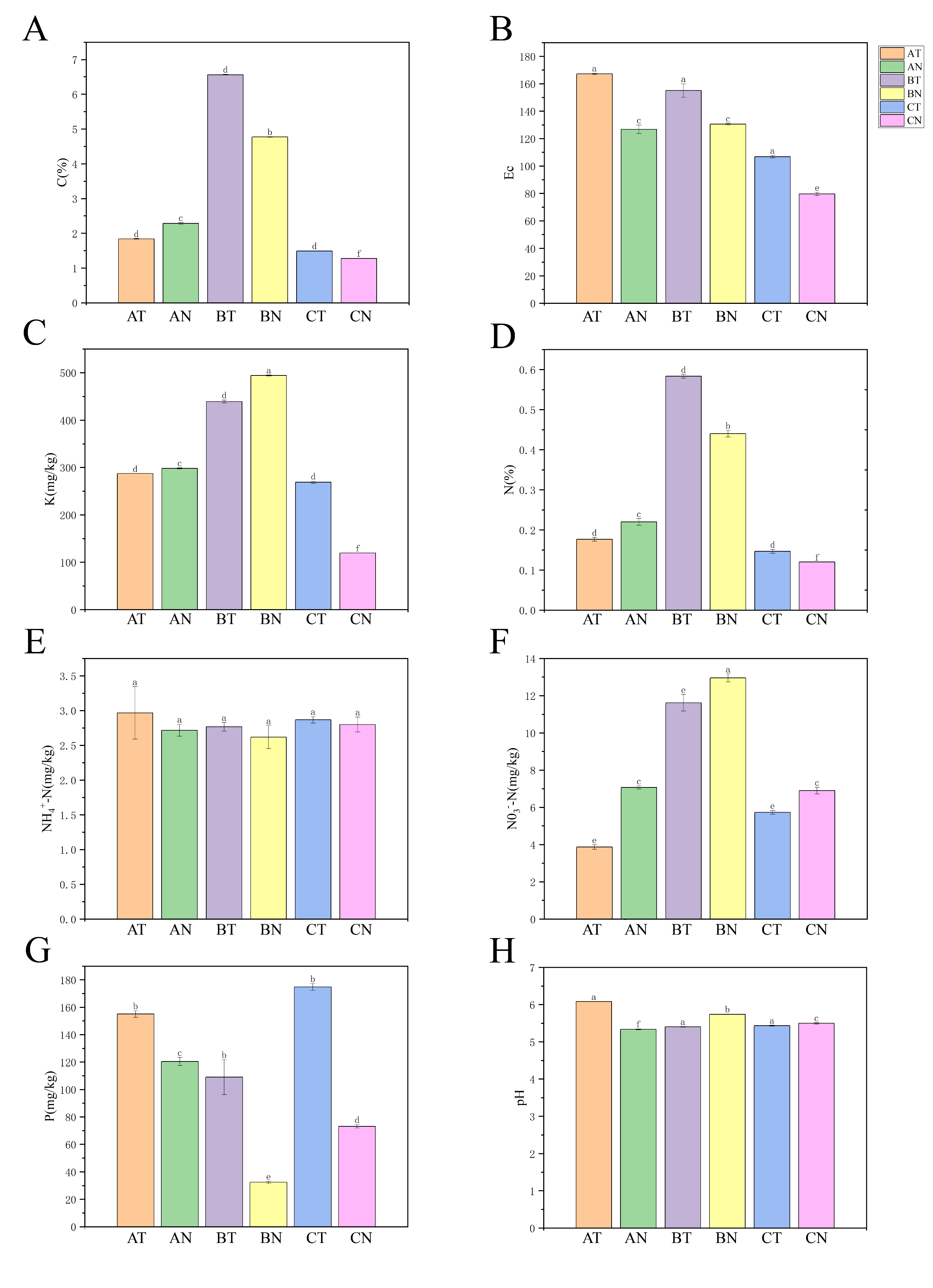


Figure S 1. Changes in soil physicochemical properties with soil fumigation. According to Duncan's new Multiple-Range test, means (N = 3) within the same period accompanied by the same letter are not statistically different (P = 0.05). AT= One year after fumigation; AN= One year after non-fumigation; BT= Two years after fumigation; BN= Two years after non-fumigation; CT= Three years after fumigation; CN= Three years after non-fumigation.


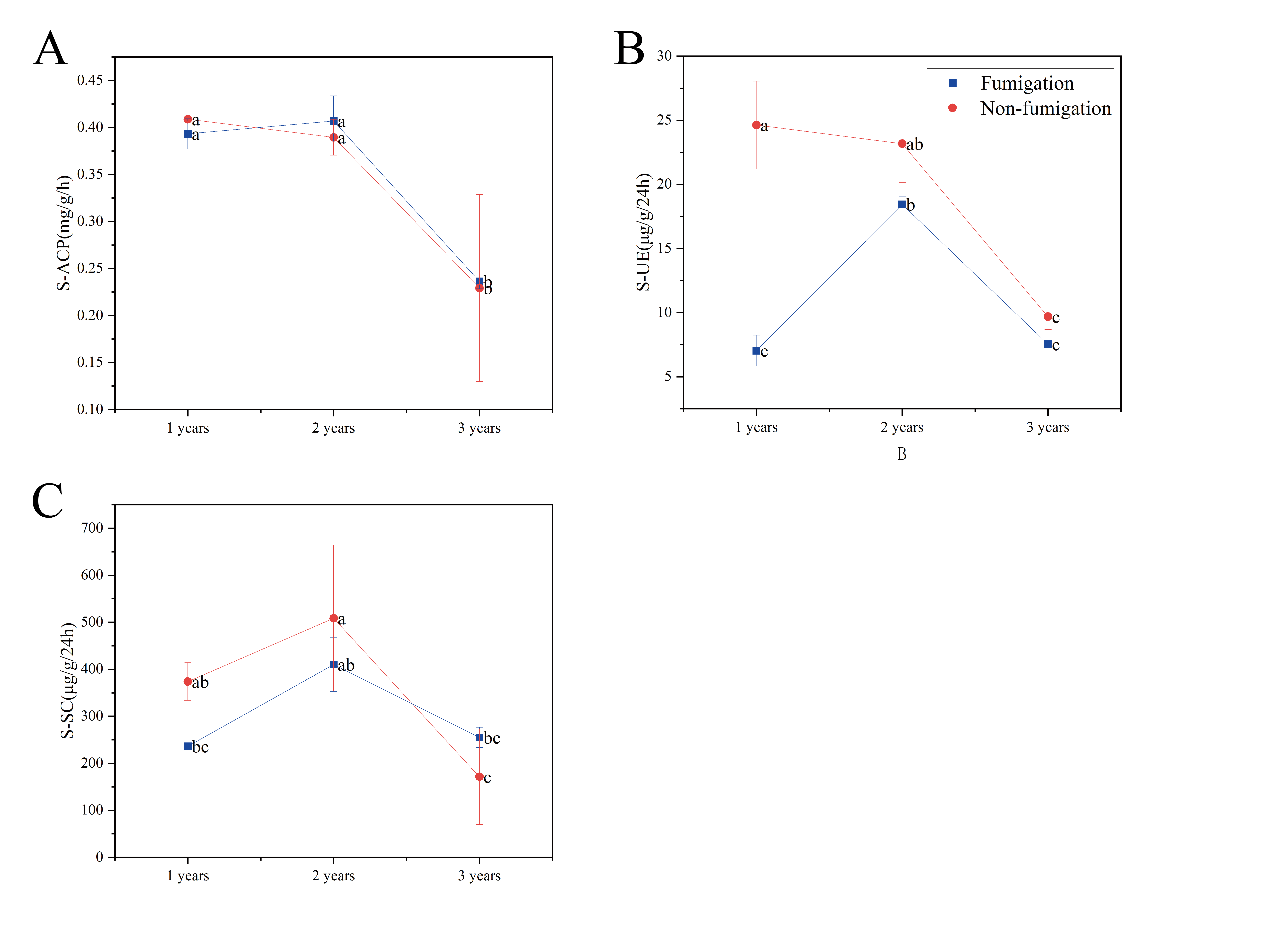


Figure S 2. Changes in soil enzyme activity with soil fumigation. According to Duncan's new Multiple-Range test, means (N = 3) within the same period accompanied by the same letter are not statistically different (P = 0.05). AT= One year after fumigation; AN= One year after non-fumigation; BT= Two years after fumigation; BN= Two years after non-fumigation; CT= Three years after fumigation; CN= Three years after non-fumigation.


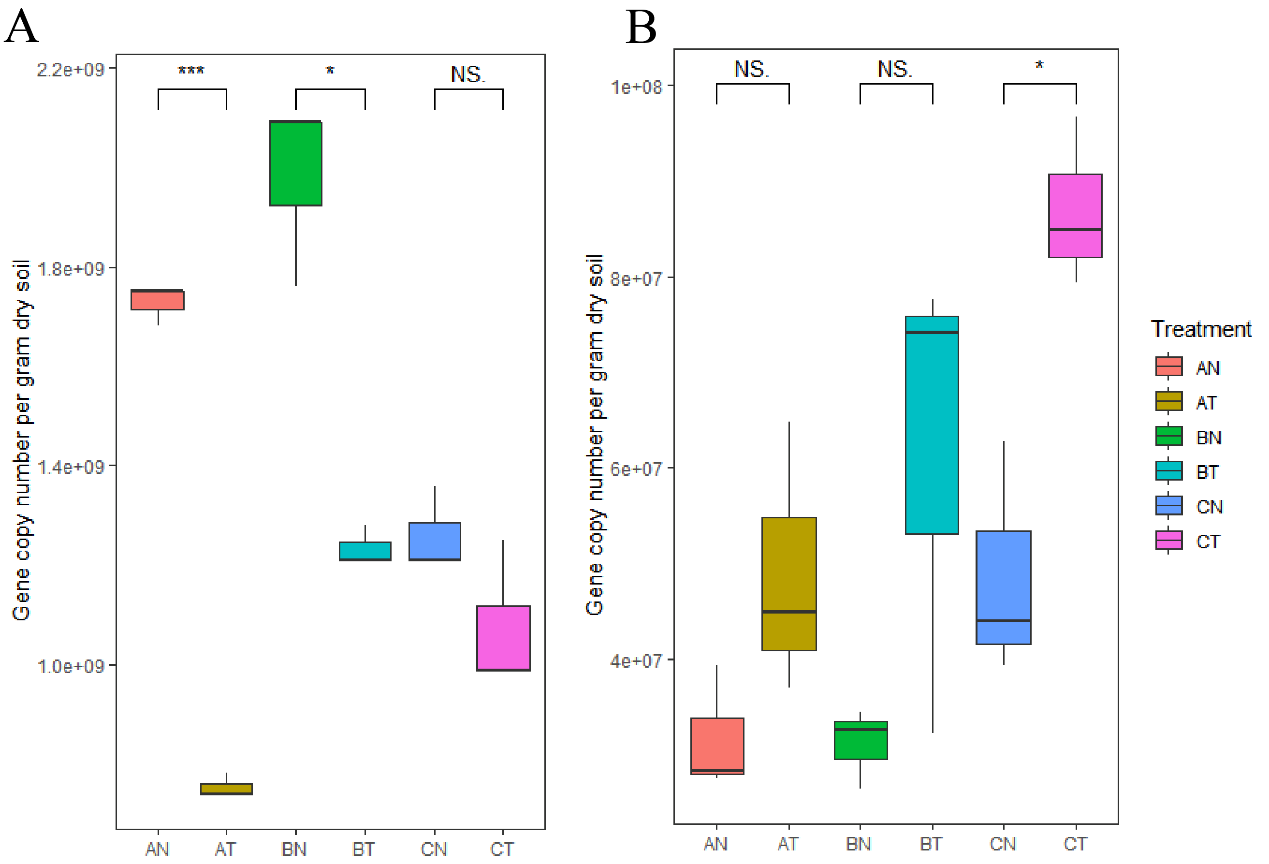


Figure S 3. Effects of on the abundance of soil bacteria(A) and fungi(B) and The number of asterisks indicates significant differences between treatments according to a one-way ANOVA. (P < 0.05): *0.01 < P ≤0.05; **0.001 < P ≤ 0.01; ***P ≤ 0.001; NS representative of P>0.05; AT= One year after fumigation; AN= One year after non-fumigation; BT= Two years after fumigation; BN= Two years after non-fumigation; CT= Three years after fumigation; CN= Three years after non-fumigation.
